# Neural stem cell heterogeneity in adult hippocampus

**DOI:** 10.1186/s13619-025-00222-4

**Published:** 2025-03-07

**Authors:** Ziqi Liang, Nuomeng Jin, Weixiang Guo

**Affiliations:** 1 State Key Laboratory for Molecular and Developmental Biology, Institute of Genetics and Developmental Biology, Chinese Academy of Sciences, Beijing, 100101 China; 2 University of Chinese Academy of Sciences, Beijing, 100093 China

**Keywords:** Adult neurogenesis, Subgranular zone, Neural stem cells, Heterogeneity, Activation, Quiescence

## Abstract

Adult neurogenesis is a unique cellular process of the ongoing generation of new neurons throughout life, which primarily occurs in the subgranular zone (SGZ) of the dentate gyrus (DG) and the subventricular zone (SVZ) of the lateral ventricle. In the adult DG, newly generated granule cells from neural stem cells (NSCs) integrate into existing neural circuits, significantly contributing to cognitive functions, particularly learning and memory. Recently, more and more studies have shown that rather than being a homogeneous population of identical cells, adult NSCs are composed of multiple subpopulations that differ in their morphology and function. In this study, we provide an overview of the origin, regional characteristics, prototypical morphology, and molecular factors that contribute to NSC heterogeneity. In particular, we discuss the molecular mechanisms underlying the balance between activation and quiescence of NSCs. In summary, this review highlights that deciphering NSC heterogeneity in the adult brain is a challenging but critical step in advancing our understanding of tissue-specific stem cells and the process of neurogenesis in the adult brain.

## Background

Historically, it was believed that the adult brain was not regarded as a stem cell system due to its perceived lack of regenerative capacity. However, Joseph Altman and his colleagues first reported in the 1960s that the generation of new neurons in adulthood occurredt in rodents, specifically in the subventricular zone (SVZ) of the lateral ventricle and the subgranular zone (SGZ) of the dentate gyrus (DG) of the hippocampus (Altman 1962, 1963; Altman and Das 1965; Altman 1969). The lifelong plasticity of the adult mammalian brain is now widely acknowledged to be significantly supported by neural stem cells (NSCs), which possess unique abilities for self-renewal and differentiation (Kempermann and Gage 1999; Christian et al. 2014; Bond et al. 2015).

During brain development, embryonic NSCs sequentially differentiate into neurons, astrocytes, and oligodendrocytes (Kennea and Mehmet 2002; Ming and Song 2011). In contrast, adult NSCs simultaneously differentiate into neurons and astrocytes in both SGZ and SVZ. Adult NSCs of the SVZ, rather than the SGZ, can generate oligodendrocytes (Menn et al. 2006; Suh et al. 2007; Bonaguidi et al. 2016). However, the potential for oligodendrocyte differentiation of adult SGZ NSCs is only achieved by overexpression of *Ascl1*(Jessberger et al. 2008) and loss of *NF1*(Sun et al. 2015a) or *Drosa*(Rolando et al. 2016). In the SVZ of the brain, NSCs migrate along the rostral migratory stream (RMS) to the olfactory bulb and solely differentiate into interneurons that are implicated in odor discrimination tasks (Lledo and Saghatelyan 2005; Alonso et al. 2006; Lledo et al. 2006). While NSCs in the SGZ differentiate into glutamatergic granule neurons locally, which are implicated in learning/memory and affective behavior (Snyder and Drew 2020). Besides the intrinsic discrepancies of NSCs in different adult neurogenic regions (Obernier and Alvarez-Buylla 2019; Borrett et al. 2022; Schiro et al. 2024), recent studies have demonstrated that even within the same region, adult NSCs are not a homogeneous population (Chaker et al. 2016; Gebara et al. 2016; Joly and Tropepe 2018; Martín-Suárez et al. 2019; Obernier and Alvarez-Buylla 2019). For instance, NSCs located in different areas of the SVZ generate distinct types of neurons postnatally, and this phenomenon is recapitulated by a cell transplantation assay (Merkle et al. 2007). Notably, given the heterogeneity within NSCs, the crosstalk between the niche and cellular intrinsic programs might generate distinct outcomes. Moreover, divergent physiological/lifestyle factors might act on distinct NSC subpopulations, thereby influencing the net neurogenesis. Since the heterogeneity of NSCs concerning regionalization and fate specification in the SVZ has been substantially summarized (Alvarez-Buylla et al. 2008; Fuentealba et al. 2015; Obernier and Alvarez-Buylla 2019), in this study, we focus on NSCs in the SGZ and thoroughly review their heterogeneity, including their origin, regional characteristics and prototypical morphology, as well as molecular mechanisms underlying their quiescent and activated status. The purpose of this review is to present current knowledge about the heterogeneity of NSCs in the SGZ, and highlight distinctions between NSC populations, throughout development, as well as within the niche. A comprehensive understanding of NSC heterogeneity will provide insights into the cellular and molecular regulation of neural development and lifelong neurogenesis, and will guide the development of novel strategies to promote regeneration and neural repair.

## Developmental heterogeneity of NSCs

Elucidating the origin of different subpopulations of adult NSCs is a challenging yet crucial step in our understanding of the process of adult neurogenesis (Berg et al. 2018). So far, three theories regarding embryonic origin have been proposed, among which the sequential model is the earliest. This model suggests that embryonic NSCs generate neurons, then glial cells, and eventually transition into the adult NSC state (Kriegstein and Alvarez-Buylla 2009). Later, the set-aside model for NSC origin in the SVZ was introduced, in which a portion of RGCs generates neurons and glia during embryogenesis while others remain dormant (from E13.5) until adulthood (Fuentealba et al. 2015; Furutachi et al. 2015). However, the models are not universally applicable. A more recent continuous model for NSC origin in the SGZ suggests that Hoxp positive-NSCs continuously generate all terminal cell types from the embryonic stage (E11.5) to adulthood (Berg et al. 2019) and collectively transition into a quiescent state in the early postnatal period (Berg et al. 2018; Berg et al. 2019; Bond et al. 2021).

In the developing brain, early signaling organizers specify anterior/posterior as well as dorsal/ventral coordinates, thereby defining major brain compartments. Within the forebrain, sonic hedgehog (Shh) and Wnt signaling instruct the ventral and dorsal telencephalon, respectively (Alvarez-Buylla et al. 2008; Rowitch and Kriegstein 2010; Fuentealba et al. 2015; Obernier and Alvarez-Buylla 2019). The NSCs in the SGZ are primarily derived from the cortical hem, one of the major hippocampal organizers (Li and Pleasure 2005), which is enriched in multiple signaling molecules (such as Wnts) (Subramanian et al. 2009). Fate-mapping analysis reveals that Axin2-positive NSCs responding to the Wnt signal emerge at embryo E11.5 and populate NSCs and surrounding niches in adult DG (Bowman et al. 2013). Moreover, a subgroup of Gli1-positive NSCs responding to the Shh signal originates from the ventral hippocampus at late gestation (E17.5) (Ahn and Joyner 2005; Li et al. 2013; Bangs and Anderson 2017). A recent study demonstrates that Axin2-positive NSCs are a subpopulation of cells dedicated to active self-renewal, while Gli1-positive NSCs represent another subpopulation of cells with a more quiescent status, which are responsive to aging and external stimuli, as well as sensitive to injury-induced action and quickly replenish NSC compartments (Luo et al. 2023b). Importantly, Axin2-positive and Gli1-positive NSCs are involved in hippocampus-dependent learning, but only Axin2-positive NSCs are engaged in buffering stress responses and depressive behavior (Luo et al. 2023b). Of note, a population of Hopx-positive NSCs in the adult DG has been found to originate at E11.5 (Berg et al. 2019). Interestingly, most Hopx-positive NSCs are not proliferating in the adult DG (Li et al. 2015). Hopx acts as an atypical homeodomain protein to regulate NSC maintenance and neurogenesis in adult hippocampus through Notch signaling (Li et al. 2015). Although it remains unclear to what extent Axin2-, Gli1- or Hopx-positive NSCs endow NSC heterogeneity in adult DG, morphogen-regulated contact-mediated signaling may contribute to the ontology of NSC heterogeneity during embryonic brain development.

## Regional heterogeneity of adult NSCs

The granular neurons in the DG are anatomically classified into dorsal dentate granule cells and ventral granule cells along the dorso-ventral axis (Amaral and Witter 1989). Beyond the differences in anatomical projections, the cells distributed along the dorso-ventral axis of the hippocampus present distinct electrophysiological characteristics. Notably, NSCs along the septo-temporal axis exhibit different characteristics. The dorsal NSCs display higher activity than the ventral NSCs (Jinno 2011). In vitro studies indicate that treatment of NSCs with norepinephrine and potassium chloride significantly increases the number of neurospheres generated in the temporal region, while neurospheres from the septal region are unaffected (Jhaveri et al. 2015). A recent study reveals that a septo-temporal molecular gradient of sfrp3 in the DG of the hippocampus may contribute to the different activities of NSCs in dorsal and ventral DG (Jang et al. 2013; Sun et al. 2015b). In addition, the adult-born neurons in dorsal DG mature at a faster rate than those in ventral DG (Jinno 2011; Piatti et al. 2011). Although previous studies have demonstrated that NSCs along the septo-temporal axis exhibits different characteristics, they have also demonstrated the existence of differences in the origin of hippocampal dorsal and ventral NSCs (Li et al. 2013), whether the functional heterogeneity of neurons in dorsal DG and ventral DG is determined by embryonic NSCs still requires further research. Furthermore, the mechanisms underlying the differences between dorsal and ventral NSCs remain an enigma.

## Heterogeneous prototypical morphology of adult NSCs

In the adult hippocampus, NSCs have distinct morphological features that distinguish them from surrounding cells. They represent a cell population with a bushy radial process, which stretches from the granule cell layer to the molecular layer and terminates with their end-feet onto the local synapses and vasculature (Moss et al. 2016). NSCs activate and generate intermediate progenitor cells (IPCs), which rapidly divide and form neuroblasts. Eventually, neuroblasts differentiate into neurons that integrate into the pre-existing circuits (Ming and Song 2011).

**Fig. 1:**
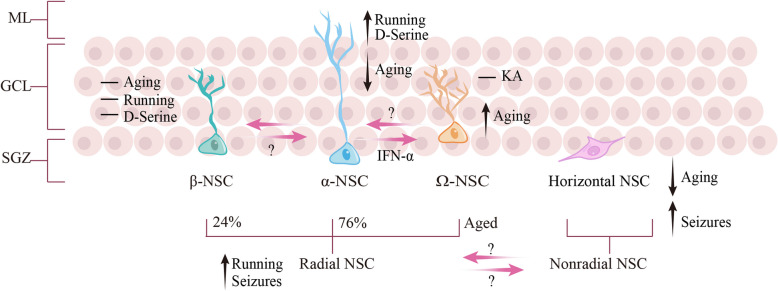
Morphologically distinct NSCs exist in SGZ of the adult hippocampus. This schematic diagram illustrates the various morphologies of NSCs located in the SGZ region. From left to right, the figure depicts radial NSCs, including β-NSCs, α-NSCs, and Ω-NSCs, as well as nonradial NSCs, specifically horizontal NSCs. The pink arrows indicate transitions between different cell types. An upward black arrow signifies active regulation of a cell type under specific conditions, while a downward arrow indicates the opposite effect. A horizontal line denotes that a cell type remains unaffected. Additionally, among Nestin and GFAP positive-NSCs, α-NSCs accounts for 76% of the radial NSCs, while β-NSCs accounts for 24%. Ω-NSCs only exists in aged conditions, and nonradial NSCs accounts for 46% of the Hes5- positive-NSCs, while radial NSCs accounts for 54%. SGZ, subgranular zone; GCL, granular cell layer; ML, molecular layer

Interestingly, there is a coexistence of two populations of radial NSCs with distinct prototypical morphological characteristics: type α NSCs (α-NSCs), which display a long primary process modestly branching into the molecular layer, and type β NSCs (β-NSCs), which have a shorter radial process highly branching into the outer granule cell layer-inner molecular layer border (Gebara et al. 2016). In addition to these distinct morphological features, α-NSCs express stem cell markers such as Nestin, Sox2, GFAP, and Prominin, while β-NSCs coexpress stem cell-specific markers (Nestin, GFAP and Prominin) and astrocyte-specific markers (GLT1 and S100β) (Gebara et al. 2016). In vivo lineage tracing by GFAP-CreER^T2^ or Nestin-CreER^T2^ mouse lines indicates that α-NSCs are bona fide NSCs that can give rise to neurons, astrocytes, and β-NSCs, while β-NSCs may represent an intermediate state in the transformation of α-NSCs into astrocytes (Gebara et al. 2016). Since both α- and β-rNSCs express GFAP and Nestin, it is worth noting that their lineage potential and relationship could not be clearly defined using GFAP-CreER^T2^ or Nestin-CreER^T2^ mouse lines alone. Subsequently, the third type of NSC, namely Ω-NSC, was discovered, which has a multibranched morphology with several primary processes, possesses proliferative capacity, and plays an increasingly significant role in the occurrence of aging (Martín-Suárez et al. 2019). Ω-NSCs do not express S100β, which distinguishes them from β-NSCs, while the presence of Nestin and LPA1 indicates a strong association with α-NSCs (Martín-Suárez et al. 2019). Except for radial-glia-like morphology, a group of quiescent NSCs exists with a horizontal morphology and expresses Hes5 and Sox2 (Lugert et al. 2010). The horizontal NSCs remain in a quiescent state and are activated under epilepsy, gradually reducing and being almost completely depleted along with age (Lugert et al. 2010). Notably, the horizontal NSCs are distinguished from IPCs, which are highly proliferative and express markers such as MCM2, Sox11, Mash1, and Tbr2, but not Hes5 (Lugert et al. 2010; Shin et al. 2015). However, the relationship between Nestin-positive horizontal NSCs and IPCs remains unclear. Although NSCs are made up of multiple subpopulations that differ in morphology, their lineage potential, relationship, and functionality are not fully understood yet (Fig. 1).

## Distinct signatures of quiescent and activated NSC

**Fig. 2:**
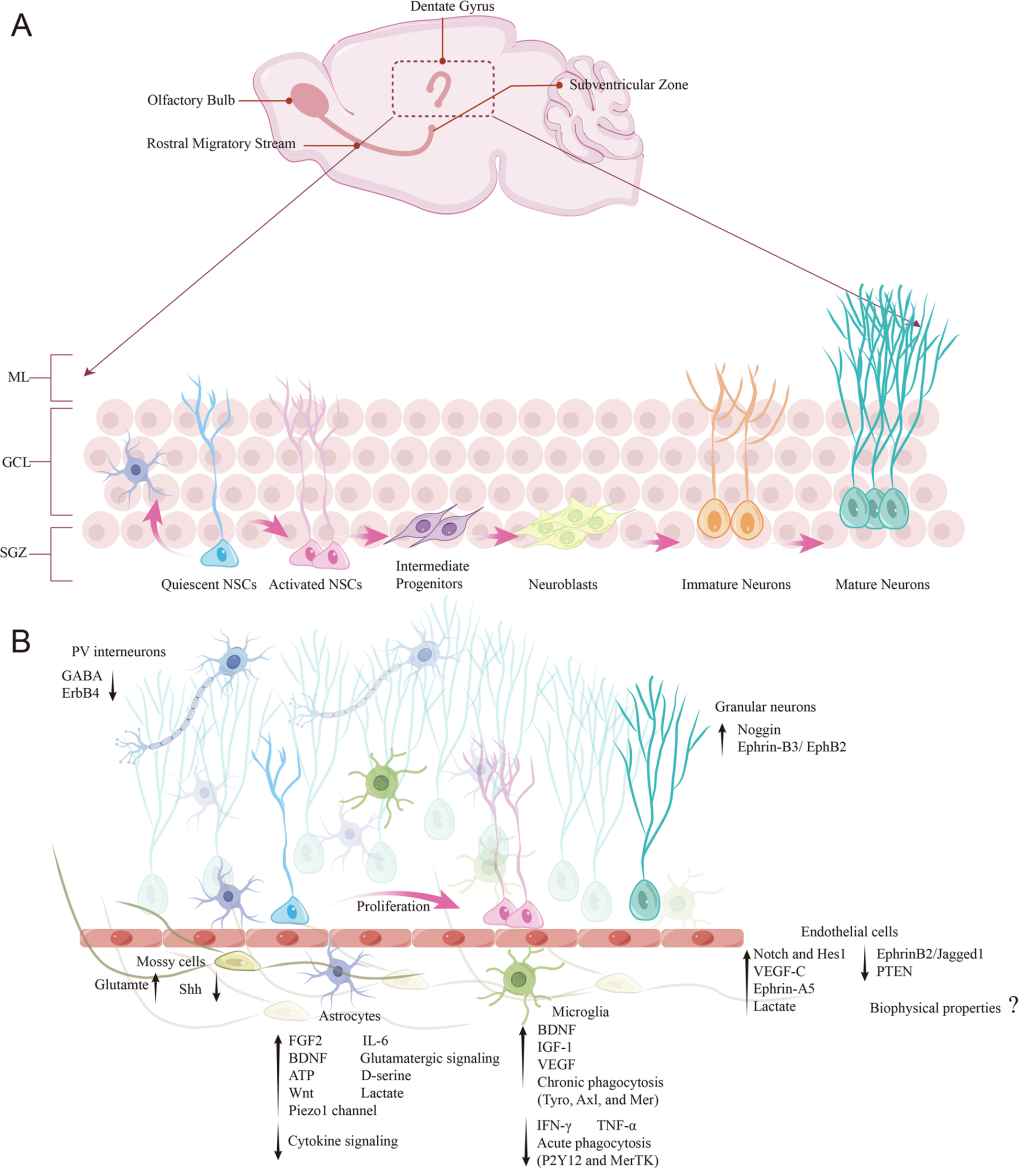
Neurogenesis and its cellular niche in the SGZ of the adult hippocampus. **A** Overview of the different stages of adult neurogenesis in the DG. New granule neurons in the DG are generated through several consecutive developmental stages. Quiescent NSCs enter an active state and subsequently generate intermediate progenitor cells (IPCs). IPCs give rise to immature dentate granule neurons, which migrate into the granule cell layer and become functionally mature neurons. In addition to producing neurons, NSCs also have the ability to produce astrocytes. SGZ, subgranular zone; GCL, granular cell layer; ML, molecular layer. **B** Schematic representation of the organization and composition of the adult hippocampal neurogenic niche. The upward black arrow indicates factors that promote the activation of NSCs, while the downward arrow represents factors that inhibit their activation

The generation of neurons relies heavily on tight control of NSC activity and neuronal differentiation (Ding et al. 2020) (Fig. 2A). Both activated and quiescent NSCs (qNSCs) maintain a balanced, long-lived pool of NSCs in the adult brain, which not only provides protection from damage but also prevents the irreversible depletion of the NSC pool (Urbán et al. 2019). Progress has been made in identifying several markers for distinguishing qNSCs and activated NSCs (aNSCs). qNSCs are marked by Id3, Id4, Hopx, Apoe, Clu, and Aldoc, while aNSCs are characterized by Ki-67, MCM2, PCNA, and Fgfr3 (Shin et al. 2015; Artegiani et al. 2017; Urbán et al. 2019; Otsuki and Brand 2020). Differences in metabolism-related genes suggest that qNSCs and aNSCs likely rely on distinct primary energy sources, with activation of qNSCs involving a shift from glycolysis to mitochondrial oxidation (Spinelli and Haigis 2018; Scandella et al. 2023). In this section, we will thoroughly summarize the cellular and molecular mechanisms underlying the balance between activation and quiescence of NSCs.

### SGZ neurogenic niche

The NSC niche in the SGZ of the hippocampus forms a highly complex network of cell-to-cell interactions. Although NSCs are regulated by autocrine factors, such as Mfge8 and PTN (Zhou et al. 2018; Tang et al. 2019; Li et al. 2023), the niche cells also play a critical role in regulating the self-renewal and differentiation potential of NSCs (Li and Guo 2021). The neurogenic niche encompasses vascular endothelial cells, astrocytes, microglia, as well as mature neurons (Fig. 2B).

NSCs in the SGZ are highly polarized, with lateral processes connecting to other radial astrocytes, a primary cilium, and a proximal domain facing the hilus. They also interact specifically with vascular endothelial cells in distinct regions (Licht et al. 2020). Vascular endothelial cells secrete vascular growth factors (VEGF and VEGF-C), which act on VEGFR3 and activate NSCs (Louissaint et al. 2002; Xiong et al. 2010; Ruan et al. 2015; Matta et al. 2021; Crouch et al. 2023; Karakatsani et al. 2023). Direct contact co-culture between endothelial cells and NSCs leads to the secretion of endothelial factors by endothelial cells, which up-regulate the Notch effector Hes1 in NSCs and promote NSC proliferation (Shen et al. 2004). In addition, vascular endothelial cells have been assumed to transport energy metabolites that affect NSC activity. A recent study demonstrates that Pten-AKT-MCT1 axis is required for vascular endothelial cells to transport excessive lactate into the blood vessel, thereby maintaining lactate homeostasis in the brain parenchyma and NSC activity (Wang et al. 2019a). Furthermore, MCT1 and MCT2 respectively control efflux and influx of lactate in NSCs, by which lactate links histone lactylation to NSC proliferation through MDM2-p53 signaling pathway (Li et al. 2025).

Astrocytes are the major components of the adult neurogenic niche (Verkhratsky et al. 2016), where they regulate NSC activity mainly by releasing various trophic factors and gliotransmitters. For example, astrocytes have been known to secrete growth factors such as FGF-2 (Song et al. 2002; Kirby et al. 2013) and Wnt (Lie et al. 2005), inflammatory factor IL-6 (Bowen et al. 2011; Wang et al. 2011), neurotrophic factor BDNF (Quesseveur et al. 2013), amino acid D-serine (Sultan et al. 2013), and gliotransmitter ATP (Cao et al. 2013), which are involved in regulation of NSC activation. Moreover, astrocytes have been known to produce and release lactate into the brain parenchyma to regulate NSC activity (Álvarez et al. 2016). In the neurogenic niche, the release of endogenous neuropeptide cholecystokinin (CCK) by dentate CCK interneurons supports neurogenic proliferation of NSCs through a dominant astrocyte-mediated glutamatergic signaling cascade (Asrican et al. 2020). Interestingly, a recent study shows that Piezo1-mediated mechanotransduction mediates astrocytic ATP release, thereby regulating NSC activation (Chi et al. 2022).

Microglia are resident immune cells in the niche. Various reports suggest that NSCs are spared from apoptosis during much of the neurogenic trajectory, and ferroptosis might also be a model of pruning in this phase of adult neurogenesis (Zhang et al. 2022; Zhang et al. 2024b). Predominantly, microglial cells phagocytose apoptotic neuroblasts and secrete a range of neurotrophic factors and cytokines, which in turn regulate the activation and differentiation of NSCs (Sierra et al. 2010; Beccari et al. 2017; Diaz-Aparicio et al. 2020). For example, chronic impairment of TAM receptor-mediated phagocytosis in microglia suppresses NSC activation. However, acute dysfunction of MerTK receptor-mediated phagocytosis leads to NSC activation (Diaz-Aparicio et al. 2020). On the other hand, microglia have been known to regulate NSC behavior via secretory factors, such as growth factors (BDNF, IGF-1, VEGF), which promote NSC activation, and inflammatory molecules (TNF-α, IFN-γ), which suppress NSC activation (Bernardino et al. 2008; Mäkelä et al. 2010; Kohman et al. 2012; Littlefield et al. 2015; Kreisel et al. 2019).

NSCs differentiate into granule neurons in the adult hippocampus, which in turn regulate NSC behavior. For example, granule neurons regulate NSC activity through direct GC-NSC contact in the local niche. The ephrin-B3 is expressed on granule neuron membrane, which functions as a molecular switch in maintaining the quiescent state of NSC via the receptor EphB2 on NSC membrane (Dong et al. 2019). Furthermore, mossy cells (MCs) constitute a major population of excitatory neurons in the adult DG and serve as a crucial component of the neurogenic niche. MCs dynamically regulate the activity of NSC through direct glutamatergic MC-NSC pathway and indirect GABAergic MC-local interneuron-NSC pathway. Moderation of MC activation increases NSC quiescence through the dominant indirect pathway, while high MC activation increases NSC activation through the dominant direct pathway (Yeh et al. 2018). In addition, MCs regulate NSC activity via secreting Shh, and specific ablation of Shh from MCs leads to overactivation of NSCs (Gonzalez-Reyes et al. 2019; Noguchi et al. 2023). As an inhibitory interneuron in the neurogenic niche, parvalbumin (PV) neurons exert long-range regulation of NSC activation either by releasing GABA neurotransmitters or modulating the ErbB4-BDNF-TrkB signaling (Song et al. 2012; Song et al. 2013; Bao et al. 2017; Zhang et al. 2018).

### Epigenetic regulation

**Fig. 3:**
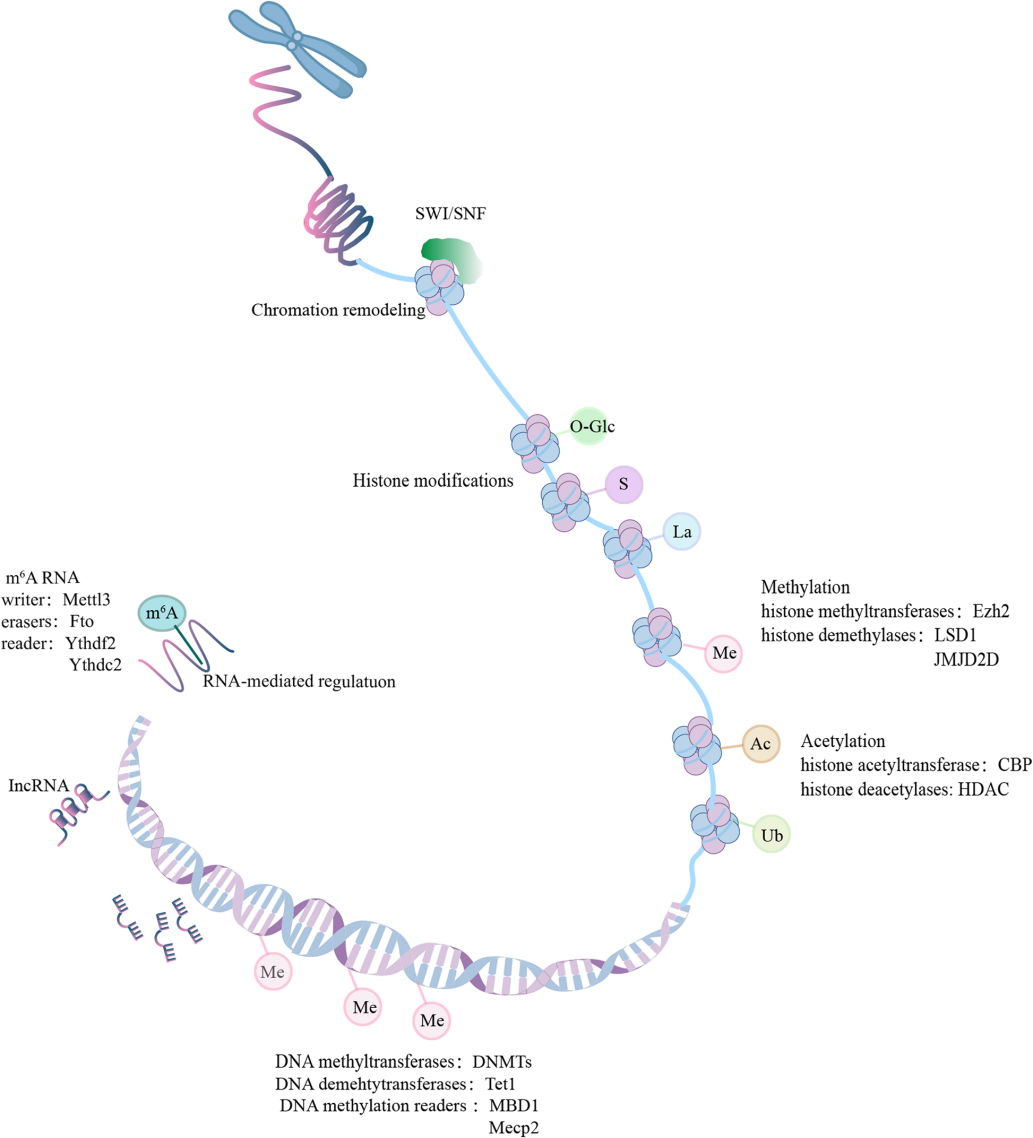
Epigenetic modifications regulating the activity of NSCs. This schematic diagram outlines the mechanisms of epigenetic regulation. It includes RNA-mediated regulation (m^6^A RNA), DNA methylation, and various histone modifications (methylation, acetylation, glycosylation, ubiquitylation, SUMOylation, and lactylation). SUMO, small ubiquitin-like modifier

In addition to extrinsic factors, intrinsic factors also actively regulate the balance between resting and activated states of NSCs (Blanchart et al. 2018; Wang et al. 2019b; Li and Guo 2021; Guo et al. 2022; Luo et al. 2023a). The roles of transcription factors and post-transcriptional regulation have been well documented (Hsieh 2012; Papadimitriou and Thomaidou 2024). It is widely believed that epigenetic mechanisms regulating dynamic changes in gene expression are crucial for NSC activity (Yao et al. 2016) (Fig. 3). In this section, we will summarize the research advancements regarding the regulation of NSC behavior by DNA methylation, m6A RNA modification, and histone modification.

DNA methylation regulates gene expression by adding methyl groups to DNA, particularly at cytosine residues in CpG dinucleotides, forming 5-methylcytosine (5mC) (Suzuki and Bird 2008; Schübeler 2015). This process is mediated by DNA methyltransferases (DNMTs) and DNA demethyltransferases (Ten-eleven translocation (TET) family). Proper DNA methylation is crucial for the maintenance of neural progenitor cells during early embryonic development and adult neurogenesis (Smith and Meissner 2013). Recent research indicates that the reduction of DNMT’s activity in rats suppresses the activation of NSC (Gou et al. 2021). As readers of DNA methylation, methyl-CpG-binding proteins (MBPs) are the main mediators of DNA methylation in regulating gene expression. Methyl-CpG binding protein 1 (MBD1), a member of the MBP family, has been shown to regulate NSC activation by modulating miR-184-Numbl axis (Zhao et al. 2003; Liu et al. 2010). Moreover, Mecp2, another MBP, governs NSC activity through miR-137-Ezh2 (a histone methyltransferase and Polycomb group (PcG) protein) pathway (Szulwach et al. 2010). As DNA demethylases, TET family enzymes are responsible for activating the erasure of the methyl group from 5mC (He et al. 2011), which is also involved in the regulation of NSC activation. For instance, Tet1 deficiency leads to hypermethylated genes involved in proliferation, thereby inhibiting NSC activation (Zhang et al. 2013). Furthermore, loss of Tet1 expression results in increased methylation of the Dll3 and Notch1 promoters, blocking Notch signaling and reducing NSC activation (Chen et al. 2021). Although the mechanisms remain unknown, Tet2 is required for NSC activation (Gontier et al. 2018).

m^6^A methylation, the most common form of mRNA modification (Meyer and Jaffrey 2014), is critical for adult hippocampal NSC activity by regulating transcription and translation. The methyltransferase complex, also known as the "writer," is responsible for methylating RNA transcripts at appropriate sites (Rana and Ankri 2016). For example, Mettl3 deficiency reduces m^6^A levels, thereby suppressing Ezh2 expression and inhibiting NSC activation (Chen et al. 2019). The demethylase Fat mass and obesity-associated gene (FTO) is identified as the main scavenging enzyme of m^6^A (Jia et al. 2011). Loss of FTO alters the expression of key components in the BDNF-TrkB pathway, thereby inhibiting the activation of NSC (Li et al. 2017). Moreover, FTO is known to govern NSC activity by modulating the Pdgfra/Socs5–Stat3 pathway (Cao et al. 2020). The m^6^A reader proteins encompass the members of the Ythdf and Ythdc families, which specifically recognize m^6^A-modified mRNAs to control the processing and translation (Patil et al. 2018). Ythdf2 deficiency activates TGF-β signaling, thereby increasing the quiescence acquisition of NSC (Zhang et al. 2024a). Moreover, Mettl3-mediated m^6^A modification of Lrp2 mRNA enhanced its stability, with Ythdc2 ensuring its translation efficiency and regulating the activation of NSC (Xu et al. 2022).

Transcription factors play a pivotal role in the regulation of NSC behavior (Shohayeb et al. 2018). Histone modifications, including acetylation, methylation, ubiquitination, phosphorylation, glycosylation, SUMOylation, and lactylation processes, facilitate the recruitment of these transcription factors to chromatin for the activation or repression of gene transcription (Bannister and Kouzarides 2011; Adam and Harwell 2020). Histone acetylation occurs on lysine residues and is catalyzed by histone acetyltransferases (HATs), while deacetylation is catalyzed by histone deacetylases (HDACs) (Yang and Seto 2007). Both HATs and HDACs play a crucial role in regulating the behavior of NSC. For example, the histone acetyltransferase CREB-binding protein (CBP) functions as an HAT, and its deficiency reduces the acetylation of histones H2B and H3, thereby decreasing the expression of genes associated with cell proliferation and suppressing NSC activity (Lopez-Atalaya et al. 2011). On the other hand, inhibition of HDAC function leads to impeded NSC activation (Foti et al. 2013). Therefore, the delicate balance between acetylation and deacetylation plays a crucial role in maintaining NSC homeostasis.

Histone methylation involves the addition of methyl groups to lysine and arginine residues. This process is mediated by histone methyltransferases (HMTs) and histone demethylases (Greer and Shi 2012) and involves the regulation of NSC activity. As an HMT, Ezh2 catalyzes the methylation of histone H3 lysine 27 (H3K27me3) and inhibits gene transcription (Margueron et al. 2009). EZH2 enhances NSC activity by inhibiting PTEN expression and activating the Akt-mTOR signaling pathway (Zhang et al. 2014). Additionally, as histone demethylases, LSD1 selectively demethylates H3K4me2 and H3K4me1, and its deficiency significantly impairs NSC activity (Sun et al. 2010). Moreover, JMJD2D regulates the methylation of H3K9 on promoters of key genes, such as Id2 and Sox2, thereby modulating NSC activity (Maitra et al. 2020).

### Cellular metabolism

**Fig. 4:**
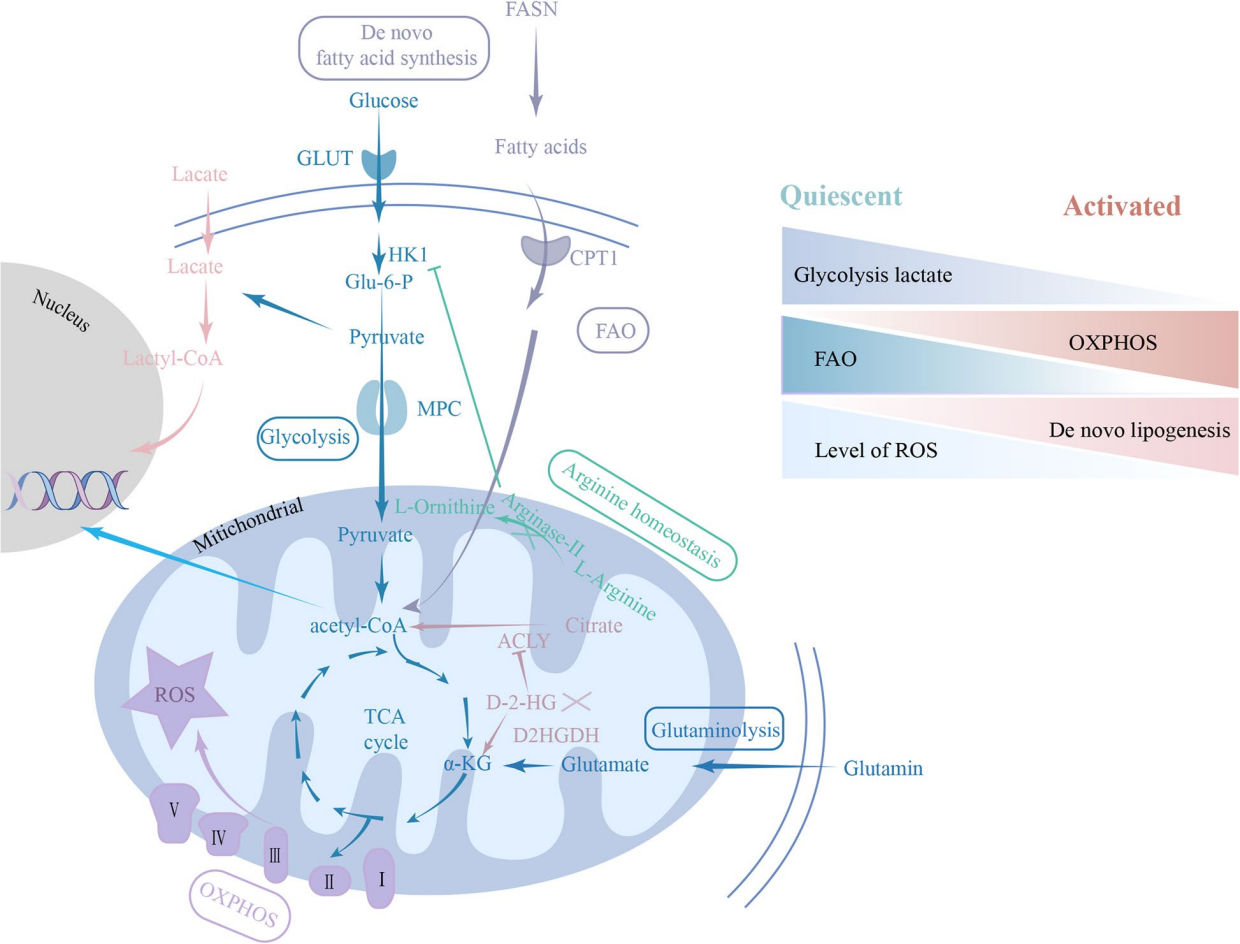
Cellular metabolism regulates the activity of NSCs. On the left, a schematic diagram summarizes the major cellular metabolic pathways discussed in this review, including glucose metabolism, lipid metabolism, and protein metabolism. Glucose undergoes glycolysis, converting to pyruvate, which can either be fermented to lactic acid (associated with histone lactate modification and subsequent secretion) or shuttled into the mitochondria for energy production via the tricarboxylic acid (TCA) cycle. The right panel provides a summary schematic of significant changes in metabolic pathways between quiescent and activated NSCs

Besides being regulated by a plethora of morphogenic signaling and transcriptional codes, neurogenesis goes hand-in-hand with metabolic alterations (Fig. 4). It is well known that glucose, lipid, and protein are the main energy sources for cells (Pang et al. 2014). At present, the research concerning lipid metabolism-regulating hippocampus adult NSCs has been summarized (Knobloch et al. 2013; Knobloch et al. 2017; Luo et al. 2023a). In this section, we summarize the studies on the impact of glucose metabolism on the activity of hippocampus adult NSCs.

Glucose is the primary energy source for the brain, accounting for about 20% of the body's total glucose consumption (Erbsloh et al. 1958; Howarth et al. 2012). qNSCs primarily rely on glycolysis for ATP production, while aNSCs undergo metabolic reprogramming to shift towards oxidative phosphorylation (OXPHOS), utilizing substrates such as pyruvate, alpha-ketoglutarate (αKG), and acetyl-CoA (Spinelli and Haigis 2018; Scandella et al. 2023). Glucose metabolism is critical for NSC behavior. For example, excessive glucose facilitates the binding of Sirt-1 to the promoter of Hes-1, thereby reducing Hes-1 expression and impairing NSC activation. However, under low glucose conditions, Sirt-1 is replaced by CREB on the Hes-1 promoter, which promotes Hes-1 expression and enhances NSC proliferation. Therefore, the glucose-mediated antagonistic interaction between CREB and Sirt-1 in regulation of Hes-1 transcription plays a role in the metabolic control of neurogenesis (Fusco et al. 2016). Glucose within the cell is phosphorylated by hexokinase (HK) to produce glucose 6-phosphate (G6P). The attachment of HK1 to mitochondria is suppressed by accumulated arginine levels, which leads to the switch from glycolysis to OXPHOS and promotes NSC overactivation (Xu et al. 2023). In addition, cellular redox states regulate the balance between the maintenance and activation of NSCs. qNSCs maintain relatively high levels of reactive oxygen species (hiROS), whereas aNSCs exhibit low levels of ROS (loROS) (Adusumilli et al. 2021).

During glycolysis, G6P is converted to pyruvate, which is transported into mitochondria by the mitochondrial pyruvate carrier (MPC). MPC inhibition increases NSC activity (Petrelli et al. 2023). Pyruvate is decarboxylated to acetyl-CoA, which enters the TCA cycle. Mitochondrial D-2-hydroxyglutarate dehydrogenase (D2HGDH) catalyzes the oxidation of D-2-hydroxyglutarate (D-2-HG) to α-KG, and α-KG is an intermediate metabolite in the TCA cycle (Kopchick and Hartline 1979). Inactivation of D2HGDH leads to D-2-HG accumulation, which inhibits NSC activation via ATP-citrate lyase (ACLY)-mediated histone acetylation (Liu et al. 2023).

### Lifestyle factor

**Fig. 5:**
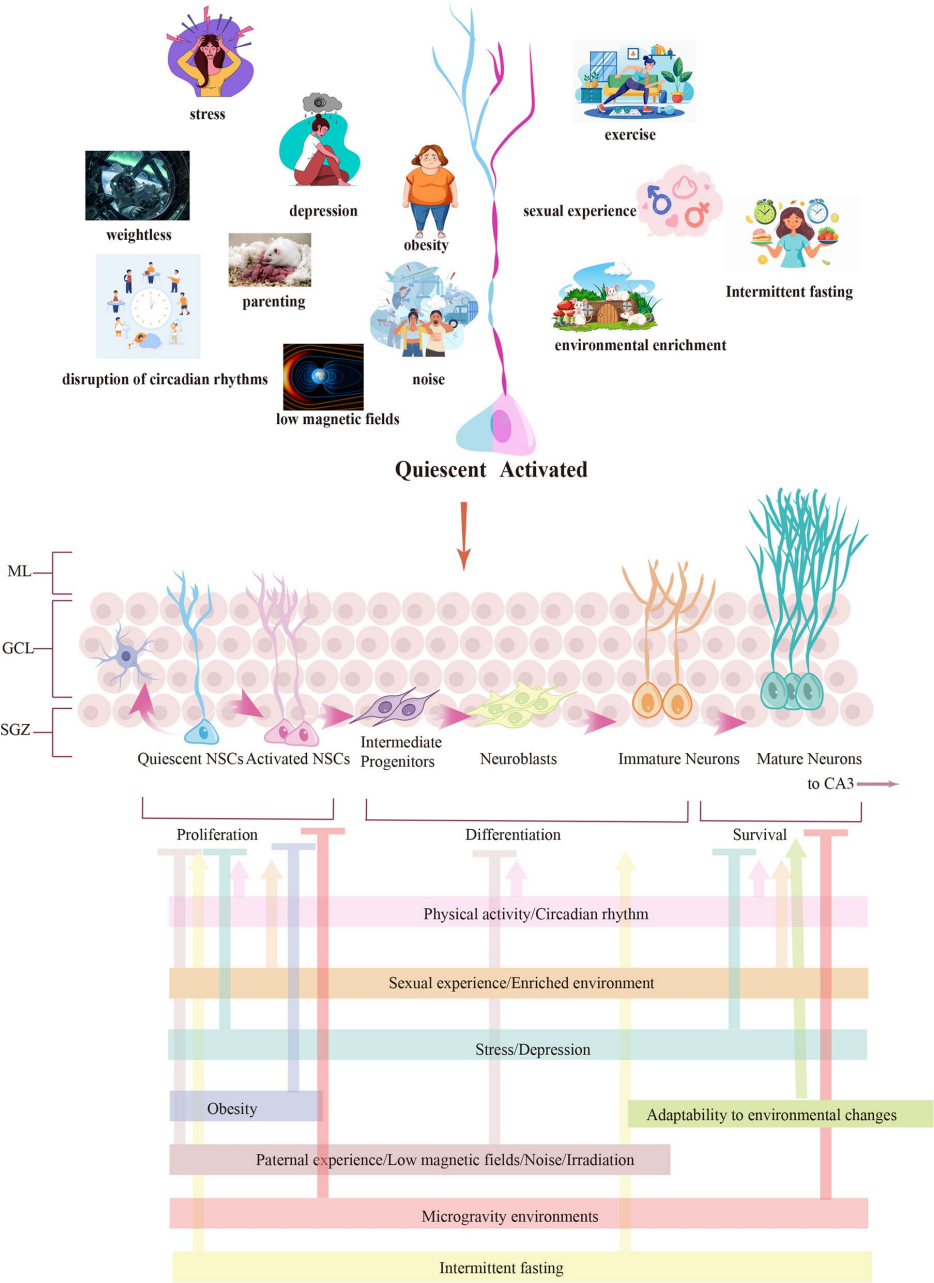
Lifestyle factors regulate the activity of NSCs. This schematic illustrates the lifestyle factors influencing SGZ NSCs. On the left are factors that promote NSC quiescence, while on the right are factors that stimulate NSC activation. Mammalian neurogenesis is regulated by many lifestyle factors. The figure demonstrates the specific influences of various lifestyles on each stage of neurogenesis, encompassing proliferation, differentiation, and neuronal survival. The arrows represent promoting factors, and the horizontal lines denote inhibitory factors

Neurogenesis in adult hippocampus is a process regulated by experience (Zhao et al. 2008) (Fig. 5). Physical exercise (Vivar et al. 2016; Adusumilli et al. 2021; Yu et al. 2021; Yi et al. 2024), sexual experience (Leuner et al. 2010; Glasper and Gould 2013), and environmental enrichment (Kempermann et al. 1997; Cope and Gould 2019; Kempermann 2019; Grońska-Pęski et al. 2021) have been demonstrated to enhance the proliferation capacity of NSCs, while stress and depression (Czéh et al. 2002; Oomen et al. 2007; Li et al. 2008; Kim et al. 2024), obesity (Park et al. 2010), and parenting (Glasper et al. 2011; Galea et al. 2014) negatively impact NSC proliferation (Opendak and Gould 2015). Recent studies suggest that low magnetic fields (HMF, intensity < 5 μT) may inhibit NSC proliferation by affecting levels of ROS (Zhang et al. 2021). Furthermore, microgravity environments encountered during space travel and the weightless effects of simulated head-down bed rest have been associated with decreased cell proliferation, potentially leading to cognitive impairments (Zhang et al. 2019). Disruption of circadian rhythms has also been shown to adversely affect neurogenesis (Liu et al. 2024). Exposure to noise (Liu et al. 2016) and irradiation (2-10 Gy) (Rola et al. 2004) increases the proportion of NSCs in the dentate gyrus region of the hippocampus that enter a quiescent state. Additional factors such as nutrition and hunger (Melgar-Locatelli et al. 2023), along with adaptability to environmental changes (Biesalski 2023), also influence NSC activity. Notably, intermittent fasting has been found to promote cell proliferation in the DG (Dias et al. 2021).

## Tools to study NSC heterogeneity

The investigation of adult NSCs is highly demanding, as they are scarce in quantity, significantly heterogeneous, and in a dynamic state (Kempermann et al. 2004; Ming and Song 2011). Conventional research on adult NSCs mainly depends on population-level analysis, which might veil the distinctive attributes of different NSC populations. Recent relevant studies have already initiated the application of single-cell analysis to discriminate among diverse NSC populations. Single-cell RNA sequencing (scRNA-seq) facilitates high-throughput analysis, revealing diverse cell types and molecular dynamics during neurogenesis across various developmental stages (Trapnell 2015; Zeisel et al. 2015; Dulken et al. 2017; Hochgerner et al. 2018; Cosacak et al. 2019). Techniques such as the Waterfall bioinformatics assay (Shin et al. 2015) have effectively captured these cellular dynamics, highlighting the heterogeneity of NSCs and their progenitors throughout development (Artegiani et al. 2017).

For the purpose of selectively observing and manipulating different types of cells within the brain, the optimal and most feasible method at present is to genetically target protein-based sensors and effectors to specific cell types (Huang et al. 2014). In mice, the Cre/lox recombination system is the most commonly used technique to mark specific cell populations with genetic elements that have specific expression patterns or loci (Gong et al. 2007; Madisen et al. 2010; Taniguchi et al. 2011; Gerfen et al. 2013). For example, Nestin-CreER^T2^, Hopx-CreER^T2^, Axin2-CreER^T2^, Gli1-CreER^T2^, Hes5-CreER^T2^ and GLAST::CreER^T2^, etc., have been successfully used to label adult hippocampal NSCs (Bonaguidi et al. 2011; Luo et al. 2023b, Lugert et al. 2010; DeCarolis et al. 2013). In fact, a specific cell type is seldom defined by single genes, but rather by the intersectional expression of multiple genes (Harris et al. 2014). Thus, many of the Cre drivers may not be sufficiently specific, thereby inadvertently resulting in inaccurate data interpretation and sometimes contradictory conclusions regarding cell fate and gene function analyses (Han et al. 2021). Recently, an intersectional genetics approach that combines orthogonal recombinases (Dre and Flpe) with Cre-lox has been developed to enhance the specificity of targeting cell subpopulations that would otherwise remain elusive with a single recombinase (Dymecki and Kim 2007; Dymecki et al. 2010; Han et al. 2021). Currently, the dual recombinase system has been successfully applied in the nervous system (Madisen et al. 2015), and it has great potential to reveal the functions and characteristics of heterogeneous NSC subpopulations.

Furthermore, given the latest advancements in microscopy technology, it is now possible to directly image the behavior of individual adult NSCs *in vivo* (Dray et al. 2015; Bottes et al. 2021; Malvaut et al. 2021). Recent studies have used two-photon microscopy to observe the two groups of NSCs specifically targeted by the Cre/loxP system marked with Gli1 and Ascl1, revealing their distinct characteristics of self-renewal potential (Bottes et al. 2021). Gli1-CreER^T2^-labeled NSCs demonstrate longer division intervals and sustained self-renewal, while Ascl1-CreER^T2^-labeled NSCs show continuous proliferation upon entering the cell cycle, eventually leading to exhaustion (Bottes et al. 2021). This approach highlights the "long-term self-renewal model" (Bonaguidi et al. 2011) represented by Gli1 and the "disposable stem cell model" (Encinas et al. 2011; Ibrayeva et al. 2021) represented by Ascl1. By combining the Cre/loxP system with two-photon microscopy, this method provides an effective tool for directly observing the behavioral heterogeneity of distinct NSC subgroups or NSCs at different ages *in vivo* (Urbán et al. 2016; Harris et al. 2021; Ibrayeva et al. 2021).

In addition, the BMP4 and FGF2 combinatorial model is the most widely used *in vitro* tool for mimicking quiescent NSCs, enabling the study of NSC activation and quiescence mechanisms (Mira et al. 2010; Martynoga et al. 2013). BMP4 alone induces a deeper quiescent state, making NSCs harder to activate (Xu et al. 2024). However, these models primarily simulate NSCs that return to quiescence after proliferation and do not effectively replicate NSCs that have never undergone division.

## Conclusions and perspectives

This article reviews and conducts an in-depth exploration of the heterogeneity of NSCs in the SGZ of the hippocampus, offering a comprehensive summary from multiple dimensions such as embryonic origin, regional functions, and morphological characteristics. We explicitly point out that NSCs in the SGZ are not a homogeneous group but are composed of a variety of interwoven and complex heterogeneous subpopulations. Particularly, proliferation serves as a core characteristic of NSCs. There are distinct signatures in the intrinsic molecular mechanisms and responses to the external environment between qNSCs and aNSCs. We emphatically discuss the homeostatic regulatory mechanisms of NSCs between the quiescent and activated states. This is a current, hot research area and plays a vital role in maintaining the NSC pool and achieving behavioral functions such as learning and memory. Regarding technical approaches, we have recapitulated multiple advanced tools for researching adult NSCs, encompassing single-cell omics technology, dual recombinase-specific labeling technology, and in vivo imaging technology. The advancement of these tools will facilitate the identification of new cell subtypes and disclose their roles in development and diseases.

Looking forward to the future, exploring the regulatory mechanisms of NSC heterogeneity is going to be a crucial research direction, which is likely to offer novel targets for the treatment of neurological disorders. An in-depth understanding of the characteristics of different NSC subpopulations, definitely helps us to develop more precise strategies to harness NSCs for neural regeneration and repair.

## Data Availability

Not applicable.

## Abbreviations

SGZ: Subgranular zone
DG: Dentate gyrus
SVZ: Subventricular zone
NSCs: Neural stem cells
RMS: Rostral migratory stream
RGCs: Radial glial cells
Shh: Sonic hedgehog
IPCs: Intermediate progenitor cells
qNSCs: Quiescent neural stem cells
aNSCs: Activated neural stem cells
CCK: Cholecystokinin
DNMTs: DNA methyltransferases
m6A: N6-methyladenosine
5mC: 5-methylcytosine
MBPs: Methyl-CpG-binding proteins
TET: Ten-eleven translocation
MBD1: Methyl-CpG binding protein 1
MeCP2: Methyl CpG binding protein 2
FTO: Fat mass and obesity-associated gene
HDACs: Histone deacetylases
CBP: CREB binding protein
HMTs: Histone methyltransferases
OXPHOS: Oxidative phosphorylation
αKG: Alpha-ketoglutarate
HK: Hexokinase
G6P: Glucose 6-phosphate
MPC: Mitochondrial pyruvate carrier
D2HGDH: D-2-hydroxyglutarate dehydrogenase
ACLY: ATP-citrate lyase
GCL: Granular cell layer
ML: Molecular layer
SUMO: Small ubiquitin-like modifier
TCA: Tricarboxylic acid cycle
MCs: Mossy cells
PV: Parvalbumin
GABA: Gamma-aminobutyric acid
